# Exercise effect on pain is associated with negative and positive affective components: A large-scale internet-based cross-sectional study in Japan

**DOI:** 10.1038/s41598-024-58340-z

**Published:** 2024-04-01

**Authors:** Kenta Wakaizumi, Yuta Shinohara, Morihiko Kawate, Ko Matsudaira, Hiroyuki Oka, Keiko Yamada, Rami Jabakhanji, Marwan N. Baliki

**Affiliations:** 1 Department of Anesthesiology, Keio University School of Medicine, 35 Shinanomachi, Shinjuku-Ku, Tokyo, 160-8582 Japan; 2 Interdisciplinary Pain Center, Keio University Hospital, Tokyo, Japan; 3 Department of Pain Medicine, Fukushima Medical University School of Medicine, Fukushima, Japan; 4 Division of Musculoskeletal AI System Development, Faculty of Medicine, The University of Tokyo, Tokyo, Japan; 5 Pain Medicine, Juntendo University Graduate School of Medicine, Tokyo, Japan; 6 Department of Anesthesiology and Pain Medicine, Faculty of Medicine, Juntendo University, Tokyo, Japan; 7 Shirley Ryan AbilityLab, Chicago, IL USA; 8 Department of Physical Medicine and Rehabilitation, Northwestern University Feinberg School of Medicine, Chicago, IL USA; 9 Center for Translational Pain Research, Northwestern University Feinberg School of Medicine, Chicago, USA

**Keywords:** Pain, Stress, Negative affect, Vigor, Emotion, Exercise, Mediation analysis, Profile of Mood States (POMS), Epidemiology, Pain

## Abstract

Pain is a global health problem that leads to sedentary behavior and tends to cause negative emotion. In contrast, exercise is widely recommended for a health promotion, while pain often worsens with physical activity. Although exercise therapy is often prescribed to people with pain, the mechanisms of exercise effect on pain remains unclear. In this study, we tried to identify a universal association factor between regular exercise and pain intensity utilizing a cross-sectional web-based survey involving 52,353 adult participants from a large national study conducted in Japan. Using principal component analysis, we uncovered a mediation model of exercise effect on pain through psychological components. Analyses were performed in half of the population with pain (*n* = 20,330) and validated in the other half (*n* = 20,330), and showed that high-frequency exercise had a significant association with reduction in pain intensity. We also found Negative Affect and Vigor, two psychological components, are fully associating the exercise effect on pain (indirect effect =  − 0.032, *p* < 0.001; association proportion = 0.99) with a dose-dependent response corresponding to the frequency of exercise. These findings were successfully validated (indirect effect of high-frequency exercise =  − 0.028, *p* < 0.001; association proportion = 0.85). Moreover, these findings were also identified in subpopulation analyses of people with low back, neck, knee pain, and the tendency of the exercise effect on pain was increased with older people. In conclusion, the effect of exercise on pain is associated with psychological components and these association effects increased in parallel with the frequency of exercise habit regardless pain location.

## Introduction

Pain is a global health problem with a high prevalence. It contributes to physical disability and reduces the motivation toward work, resulting in loss of productivity represented by presenteeism and absenteeism^1,2^. People suffering from pain tend to exhibit sedentary behavior and negative emotions such as depression and anxiety^3,4^, which significantly affect their quality of life and daily living. Exercise on the other hand, is beneficial for health, and moderate exercise habits are recommended for improving lifestyle diseases. World Health Organization defines health as “a state of complete physical, mental, and social well-being and not merely the absence of disease or infirmity”. Regular physical activity prevents several health problems, including reduced motor function, frailty, and cognitive dysfunction^5^. In addition, exercise is also known to effectively prevent and treat anxiety, depression, and stress-related symptoms, and is known to improve mental health^6^.

However, pain can be worsened with physical activity, and given the psychological effects of pain mentioned above, people with pain symptoms find it difficult to maintain the habit of regular exercise. In fact, factors such as lack of social support, decreased physical activity, decreased physical function, depression/anxiety, and decreased self-efficacy have been reported to inhibit the acquisition of exercise habits^7,8^. However, it is a well-known fact that a single session of exercise can result in reduced pain intensity and a higher threshold of pain^9^. This phenomenon is referred to as exercise-induced hypoalgesia (EIH). Although the effect of EIH resulting from a single bout of exercise is not sustained, regular exercise can exert pain relief effects in patients with chronic pain, and also prevent the transition to chronic pain^10^. It has also been reported that increasing the frequency of exercise per week is likely to result in pain relief^11^.

Epidemiological studies also support the fact that physical activity possibly prevents the development of chronic pain. A population-based study from Norway showed that people who indulged in moderate leisure-time activity one to three times per week, were significantly less likely to experience chronic musculoskeletal pain compared to those without any leisure-time activity^12^. Thus, there definitely exists a relationship between pain and regular physical activity. A review paper has suggested the involvement of the central nervous system in the effect of exercise on pain in patient populations^10^. However, there is little evidence for emotional involvement of the pain modulation effect caused by regular exercise. Therefore, we hypothesized that emotional factors associated with development of chronic pain such as stress, negative emotions, and positive emotions are involved in the relationship between pain and exercise habits and conducted a mediating analysis using data from a large-scale epidemiological survey. We also investigated on the effect of frequency of exercise habits, the site of painful symptoms, and background factors on this relationship.

## Methods

### Ethical concerns

The present study was conducted in accordance with the tenets of the declaration of Helsinki, 1975, and its revision in 2013, as well as an ethical guideline for medical and health research involving human subjects that has been issued by the Japanese Ministry of Health, Labor, and Welfare. The Japanese survey study was approved by the University of Tokyo Research Ethics Committee (approval number: 2018132NI).

### Study population

A web-based epidemiological survey was conducted for the general Japanese population, aged 20–64 years in February 2015 as described previously^13^. After an informed consent was obtained from all the study participants, 52,353 people voluntary responded to the survey, and 653 individuals suffering from cancer were excluded from this study. A total of 51,701 participants were included in the present study; mean age and standard deviation were 42.7 and 12.1 years respectively, and the proportion of women was 49.9%.

### Measures

In the web-based epidemiological study, participants were asked to choose one among the following four levels of frequency of at least 30-min exercise habits over the past year; high frequency (at least twice per week), moderate frequency (once per week), low frequency (a couple of times per month), or no exercise at all. Average pain intensity in the past four weeks was measured using the numerical rating scale (NRS)^14^, where “0” corresponded to no pain and “10” indicated worst possible pain. All individuals also completed the 11-scale subjective stress questionnaire (0: no stress, to 10: worst imaginable stress), 11-scale subjective current health condition questionnaire (0: worst, to 10: best), and the Profile of Mood States (POMS)–Brief Form, Japanese version regarding the levels of stress, health, and mood over the past four weeks^15^. The POMS is a 30-item questionnaire assessing the mood of the individuals, based on six mood construct domains as follows: tension–anxiety, depression–dejection, anger–hostility, fatigue, confusion, and vigor. Each item is rated on a five-point scale, and the score for each domain ranges from 0 to 20; higher scores indicate more disturbances, except for the vigor domain. Individuals who reported an educational level lower than high school degree were classified as the low education group. The following characteristics were investigated as well: body mass index (BMI), smoking status (current smoker or non-smoker), marital status (married, never married, divorced, or widowed), living status (alone or with family), living area (47 Japanese prefectures), sleep duration (< 5 h; ≥ 5, < 6 h; ≥ 6 h; < 7 h; ≥ 7 h, < 8 h; ≥ 8 h; < 9 h; or ≥ 9 h). One-way analysis of covariance (ANCOVA), chi-squared test, and Kruskal–Wallis test were used for comparing the demographic characteristics and behavioral measures among people without pain (NRS = 0), those with mild pain (NRS = 1–3), and those with moderate-to-severe pain (NRS ≥ 4). We performed post-hoc analyses between people with painful condition (mild and moderate-to-severe pain) and those without pain as a control using the Dunnett’s method for parametric multiple comparison, the Steel’s method for nonparametric multiple comparison, and chi-squared test for categorical data. Participants reported pain duration (< 3 or ≥ 3 months) and painful sites (multiple answers allowed out of three major pain sites: low back, neck, and knees). Chi-squared test was used to compare the pain characteristics between individuals with mild pain and those with moderate-to-severe pain.

### Principal component analysis of psychological measures

A principal component analysis (PCA) was performed with orthogonal rotation to the subjective stress and the five subscales were assessed on the basis of POMS to reduce the dimensionality of psychological measures and obtain more reliable effective variables generated by the central nervous system. Criteria of > 1 eigenvalue and > 10% explained variance were used for determining the principal components.

### Multivariable regression models of pain intensity

Exercise habit (model 1) and the psychological components identified by the PCA (model 2) were analyzed using multivariable regression models of pain intensity, with adjustment for age, sex, BMI, low education, smoking status, marital status, living status, living area, sleep duration, and pain duration. The model included three levels of exercise frequency. The psychological components derived from the PCA were also incorporated in the model. Standardized regression coefficient (std-β) was calculated as a comparable value. The F-test and adjusted R-square were used for comparing the improvement of model fitting between the first and second models. Associations of the psychological components to pain intensity in the subpopulations with low back, neck, knee, and multi-site pain were analyzed using the second model.

### Development of a mediation model for the influence of exercise on pain

The participants with pain (*n* = 40,660) were randomly divided into two groups, termed Discovery (*n* = 20,330) and Validation (*n* = 20,330), and the mediation model of the effect of exercise on pain intensity was examined through the psychological components in these two groups. Bootstrap multivariable regression analyses were used with 10,000 permutations under adjustments for age, sex, BMI, low education, smoking status, marital status, living alone, living area, sleep duration, and pain duration. The two central components derived from the PCA were theoretically independent, making the construction of a parallel mediation model possible. First, the effect of three frequent levels of exercise habit were examined and compared with no exercise in the Discovery group. The magnitude of path effects was represented by std-β, and the cumulative indirect effect was computed as a summation of individual indirect effects of the first (*a*_1_ × *b*_1_) and second (*a*_2_ × *b*_2_) components. The mediation proportion was calculated as the cumulative indirect effect out of the total effect. An identical mediation analysis was then performed in the Validation group to test the reproducibility of the model.

### Mediation analysis for effect of exercise on subjective health

Subjective health was applied to the mediation model of the exercise effect through the identified components in the Discovery group, instead of pain intensity. A two-tailed, unpaired t-test was performed under a null hypothesis that both proportions were indifferent after log-transformation of the proportional values, in order to demonstrate a difference in the mediation proportion from the model of pain intensity.

### Sub-population studies of the developed mediation model

The mediation model was also applied to the subpopulations corresponding to the pain sites and impacts: low back, neck, knee, and multi-site. In addition, the cumulative indirect and total effects were computed in subpopulations stratified according to participant characteristics, including age (20–29, 30–39, 40–49, 50–59, and 60–64 years), sex (women and men), BMI (< 20, ≥ 20 and < 25, and ≥ 25 kg/m^2^), educational level (low and high), smoking status (current and the others), living status (alone and with family), marital status (married and single including divorced and widowed), and pain duration (< 3 or ≥ 3 months).

### Statistical software and map visualization

All statistical tests were two-sided. MATLAB 2016a was used for mediation analyses. PCA, multiple regression analyses, and the other statistical analyses were performed using JMP Pro version 13.2 (SAS Institute, Cary, NC).

## Results

### Pain prevalence, severity, and associated demographics and behavioral characteristics

Of the 51,701 participants, 11,041 (21.4%) reported no pain, 25,119 (48.6%) reported mild pain (NRS = 1–3), and 15,541 (30.1%) reported moderate-to-severe pain (NRS ≥ 4). Relative to the other groups, the group with moderate-to-severe pain included people who were elderly. Also, a greater proportion of this group consisted of women. The other characteristics of participants in this group were: low educational level, currently smoking, short sleep duration (< 6 h), increased BMI, subjective stress, tension–anxiety, depression–rejection, anger–hostility, fatigue, and confusion. This group also included a lower proportion of participants with high-frequency exercise habit (more than twice per week), lower subjective health, and lower vigor (Table 1). In addition, the group with moderate-to-severe pain included a more participants with persistent pain (pain duration ≥ 3 months) and multi-site pain versus the group with mild pain.

**Table 1:** Demographic characteristics and behavioral measures of individuals with and without pain.

	No pain		Mild pain		Moderate-to-severe pain		*p*-value
Subjects, *n* (%)	11,041	(21.4)	25,119	(48.6)	15,541	(30.1)	–
Age (years), mean (SD)	40.1	(12.2)	43.5	(12.2) ***	43.0	(11.6) ***	< 0.001 †
Women, *n* (%)	4993	(45.2)	12,348	(49.2) ^###^	8438	(54.3) ^###^	< 0.001 ¶
BMI (kg/m^2^), mean (SD)	22.0	(3.6)	22.3	(3.7) ***	22.5	(4.0) ***	< 0.001 †
Low education, *n* (%)	6019	(54.5)	14,444	(57.5) ^###^	9762	(62.8) ^###^	< 0.001 ¶
Smoking, *n* (%)	2537	(23.0)	6032	(24.0) ^#^	4162	(26.8) ^###^	< 0.001 ¶
Living alone, *n* (%)	8947	(81.0)	20,517	(81.7)	12,741	(82.0)	0.14 ¶
Subjective stress, mean (SD)	3.17	(2.45)	4.04	(2.07) ***	5.77	(2.06) ***	< 0.001 †
Subjective health, mean (SD)	7.91	(2.54)	7.15	(2.16) ***	6.09	(1.87) ***	< 0.001 †
Profile of mood states (POMS), mean (SD)							
Tension–anxiety	2.86	(3.88)	4.16	(3.96) ^∫∫∫^	6.51	(5.03) ^∫∫∫^	< 0.001 ‡
Depression–dejection	2.16	(3.62)	3.10	(3.77) ^∫∫∫^	5.33	(4.99) ^∫∫∫^	< 0.001 ‡
Anger–hostility	2.41	(3.52)	3.49	(3.69) ^∫∫∫^	5.52	(4.79) ^∫∫∫^	< 0.001 ‡
Fatigue	3.09	(4.11)	4.65	(4.23) ^∫∫∫^	7.66	(5.49) ^∫∫∫^	< 0.001 ‡
Confusion	4.73	(2.73)	5.40	(2.89) ^∫∫∫^	7.17	(3.79) ^∫∫∫^	< 0.001 ‡
Vigor	4.69	(4.69)	5.03	(4.07) ^∫∫∫^	4.34	(3.87)	< 0.001 ‡
Exercise habit, *n* (%)			_###_		_###_		
High frequency	2206	(20.0)	4979	(19.8)	2624	(16.9)	< 0.001 ¶
Moderate frequency	1070	(9.7)	2761	(11.0)	1542	(9.9)	
Low frequency	807	(7.3)	2283	(9.1)	1318	(8.5)	
No regular exercise	6958	(63.0)	15,096	(60.1)	10,057	(64.7)	
Marital status, *n* (%)			_###_		_###_		
Married	5710	(51.7)	14,168	(56.4)	8477	(54.5)	< 0.001 ¶
Never married	4694	(42.5)	9104	(36.2)	5659	(36.4)	
Divorced	523	(4.7)	1577	(6.3)	1168	(7.5)	
Widowed	114	(1.0)	270	(1.1)	237	(1.5)	
Sleep duration, n (%)					_###_		
< 5 h	1127	(10.2)	2447	(9.7)	2743	(17.7)	< 0.001 ¶
≥ 5, < 6 h	3617	(32.8)	8314	(33.1)	5535	(35.6)	
≥ 6, < 7 h	3817	(34.6)	8692	(34.6)	4262	(27.4)	
≥ 7, < 8 h	1866	(16.9)	4266	(17.0)	1863	(12.0)	
≥ 8, < 9 h	455	(4.1)	1070	(4.3)	663	(4.3)	
≥ 9 h	159	(1.4)	330	(1.3)	475	(3.1)	
Pain intensity, mean (SD)	–		1.90	(0.81)	5.67	(1.45)	–
Pain duration ≥ 3 months, *n* (%)	–		12,039	(47.9)	10,020	(64.5)	< 0.001 ¶
Pain site							
Low back	–		2086	(8.3)	840	(5.4)	< 0.001 ¶
Neck	–		6009	(23.9)	2031	(13.1)	
Knees	–		844	(3.4)	290	(1.9)	
Multi-site	–		2752	(11.0)	5293	(34.1)	

Individuals with pain were divided into those with mild pain intensity (numerical rating scale [NRS] = 1–3) and those with moderate-to-severe pain (NRS ≥ 4). SD: standard deviation, BMI: body mass index. †one-way ANOVA, ¶ chi-squared test, ‡Kruskal–Wallis test. ***p < 0.001 on the post-hoc analysis using Dunnett’s parametric multiple comparison, ^∫∫∫^p < 0.001 on the post-hoc analysis using Steal’s nonparametric multiple comparison, ^#^*p* < 0.05 and ^###^*p* < 0.001 on the post-hoc analysis using chi-squared test.

### Relationships of pain intensity with exercise and behavioral characteristics

**Figure 1:**
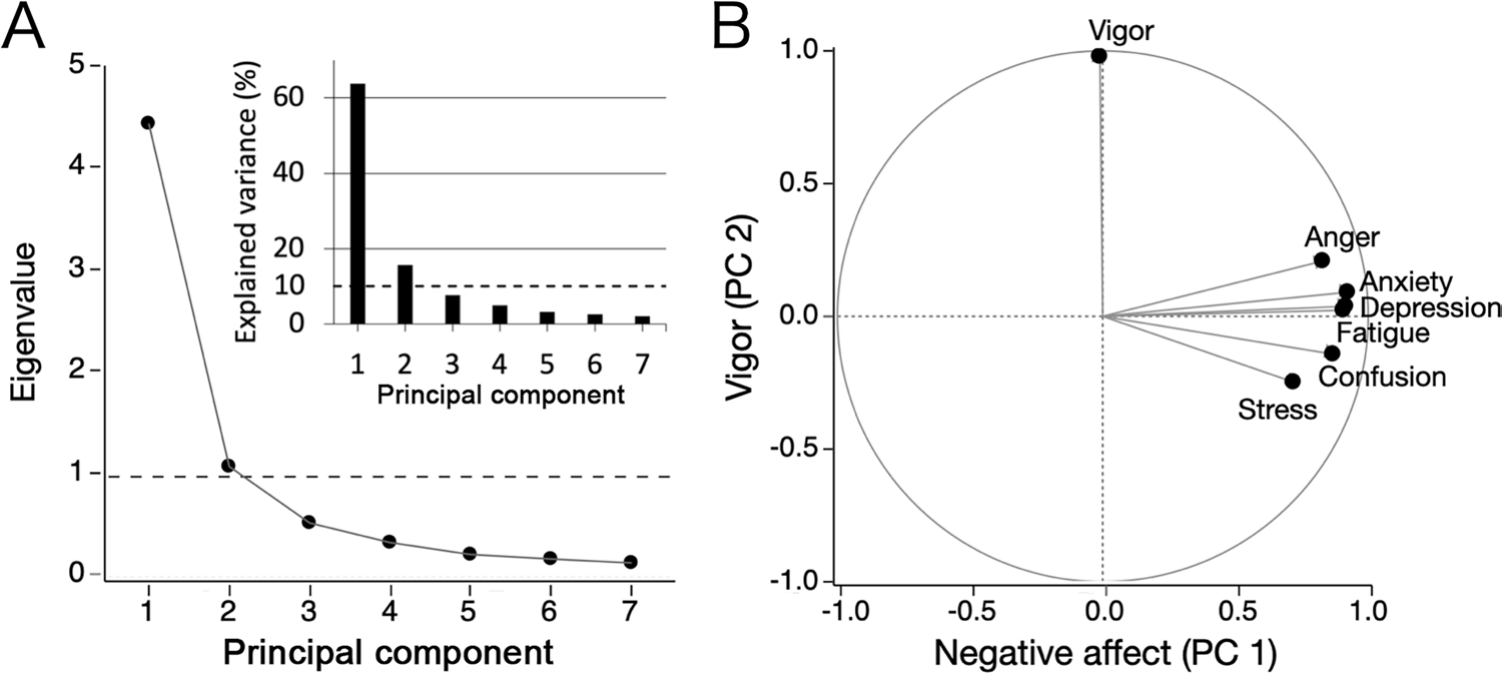
Principal component analysis of the Subjective Stress and five subscales of the POMS (*n* = 51,701). **(A)** A screen plot of eigenvalues and a bar graph of explained variances corresponding to the possible principal components. The number of components was determined by the criteria of > 1 eigenvalue and > 10% variance explained. **(B)** Loading plot of the measures for the identified two principal components.

First and foremost, based on multivariable regression analysis, a significant association was observed between reduced pain intensity and high-, moderate-, and low-frequency exercise habits compared with no exercise, with the other parameters such as age, sex, BMI, low education, smoking status, marital status, living status, living area, sleep duration, and pain duration as controls (Table 2, model 1). Two principal components were identified using PCA, which was used to reduce the psychological variables. The first principal component (PC1), which met the criteria with 4.47 eigenvalue and 63.8% of explained variance (Fig. 1A), was named Negative Affect, because variables with high loadings above 0.7 for the PC1 included subjective stress, tension-anxiety, depression-dejection, anger-hostility, fatigue, and confusion (Fig. 1B). The second principal component (PC2) with 1.09 eigenvalue and 15.6% of explained variance (Fig. 1A) was mainly composed of vigor, a domain of the POMS. Loading of vigor for the PC2 was 0.98, and the other variables showed small loadings for it (Fig. 1B). These two psychological components were significantly associated with pain intensity (Table 2, model 2), as well as in the four subpopulations of back, neck, knee, and multi-site pain (Table 3). On the other hand, significant effects of exercise habits, which were identified in the model in the absence of the psychological components, disappeared when Negative Affect (PC1) and Vigor (PC2) were included, implying that these components may be associated with the effect of exercise on pain.

**Table 2:** Multivariable regression models of pain intensity (*n* = 40,680).

Independent variable	Model 1			Model 2		
	Std-β	95% CI (LL, UL)	*t*-value	Std-β	95% CI (LL, UL)	t-value
High-frequency exercise	− 0.033	(− 0.042, − 0.023)	− 6.62***	− 0.004	(− 0.013, 0.005)	− 0.95
Moderate-frequency exercise	− 0.023	(− 0.032, − 0.013)	− 4.68***	− 0.007	(− 0.016, 0.002)	− 1.46
Low-frequency exercise	− 0.013	(− 0.022, − 0.003)	− 2.65**	− 0.007	(− 0.016, 0.002)	− 1.59
Negative affect (PC1)				0.360	(0.351, 0.369)	77.96***
Vigor (PC2)				− 0.115	(− 0.124, − 0.106)	− 25.58***
Adj-R^2^ (Δadj-R^2^)		0.085		0.214	(0.128)	
F (ΔF)		60.4***		168.3***	(3307.6***)	

Regression models were adjusted for age, sex, BMI, low education, marital status, living alone, living area, smoking status, sleep duration, and pain duration; **p < 0.01, ***p < 0.001. Std-β = standardized regression coefficient, CI = confidence interval, LL = lower limit, UL = upper limit, adj-*R*^2^ = adjusted *R* square.

**Table 3:** Associations of negative affect (PC1) and vigor (PC2) with pain intensity in sub-populations with different pain conditions.

	Negative Affect (PC1)		Vigor (PC2)		*F*	adj-*R*^2^
	Std-β	95% CI (LL, UL)	Std-β	95% CI (LL, UL)		
Back pain (*n* = 2926)	0.308***	(0.273, 0.343)	− 0.115***	(− 0.149, − 0.081)	10.3***	0.173
Neck pain (*n* = 8040)	0.295***	(0.274, 0.316)	− 0.111***	(− 0.132, − 0.090)	23.7***	0.157
Knee pain (*n* = 1134)	0.295***	(0.236, 0.353)	− 0.131***	(− 0.188, − 0.074)	3.9***	0.143
Multi-site pain (*n* = 8045)	0.369***	(0.348, 0.389)	− 0.122***	(− 0.142, − 0.102)	32.4***	0.205

Multivariable regression analyses were controlled for age, sex, BMI, low education, marital status, living alone, living area, smoking status, sleep duration, pain duration, and regular exercise (same model as model 2 in Table 2); ***p < 0.001. The number of participants was represented in parentheses. Std-β = standardized regression coefficient, CI = confidence interval, LL = lower limit, UL = upper limit, adj-*R*^2^ = adjusted *R* square.

### Psychological effects associated with effect of exercise on pain

**Figure 2:**
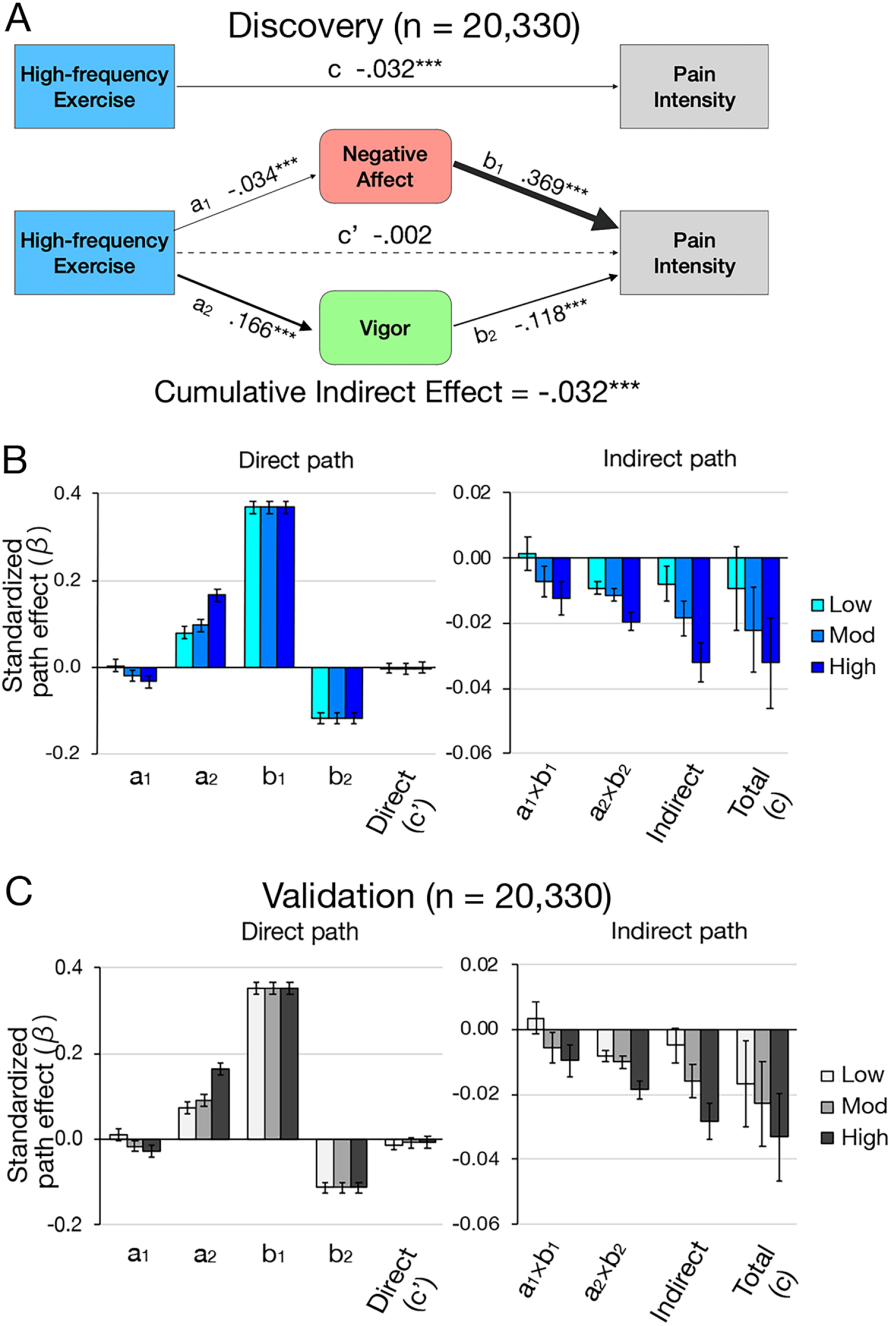
Dose–response of the full association effect of exercise on pain intensity through the psychological components. **(A)** Mediation model and computed path effects of the high-frequency exercise in half of our participants, the Discovery group (*n* = 20,330). The cumulative indirect effect, an overall psychological effect, was − 0.032 (95% confidence intervals [CI]; − 0.038 to -0.026) regarding high-frequency exercise on pain intensity, while the direct effect (c’) was nearly zero (95% CI; − 0.015 to 0.011). The thickness of the path represents the absolute value of the effect, and the dot line indicates statistical indifference from zero. ***p < 0.001. **(B)** Dose-dependent increase of absolute path effects of the mediation model in the Discovery group. The indirect and total effects increased with three levels of exercise: low, moderate (mod), and high frequency. (**C)** Replication of the full mediation model and frequency-dependent increase of the absolute path effects in the other half of the participants, the Validation group (*n* = 20,330). Bootstrap mediation analyses were performed with 10,000 permutations. Error bars represent 95% CI.

High-frequency exercise significantly decreased Negative affect (PC1) and increased Vigor (PC2), and each indirect effect to pain intensity showed significance in terms of both psychological components (Fig. 2A). The direct effect of high-frequency exercise on pain was nearly zero, and the cumulative indirect effect was nearly equal to the total effect (association proportion = 0.99), implying that the effect of exercise on pain reduction was fully associated with Negative affect (PC1) and Vigor (PC2). Furthermore, dose-dependent responses were identified in the total and indirect effects, as well as the effects of exercise on each psychological component corresponding to the frequency of exercise habit (Fig. 2B and Supplementary Table 1). In addition, even in people with low- and moderate-frequency exercise, the cumulative indirect effects were nearly equal to the total effects, suggesting full association effects. The total and indirect effects increased in parallel with the frequency of exercise habit. The dose–response and the full association effect were replicated in the validation group (Fig. 2C and Supplementary Table 2).

### Psychological association effects of subjective health with exercise

A similar analysis to examine the association effects of Negative affect (PC1) and Vigor (PC2) on subjective health was performed. Overall, the association proportion of these psychological components was observed to be lower on subjective health (0.52) than on pain intensity, whereas indirect effects were significantly high (Supplementary Fig. 1, Supplementary Table 3). Furthermore, statistically significant differences of any standardized regression coefficients between the mediation models on pain intensity and subjective health were absent, although direct effect of high-frequency exercise on subjective health was significantly higher than that on pain intensity (Supplementary Table 4).

### Robustness of the mediation model across different pain conditions and demographic characteristics

**Figure 3:**
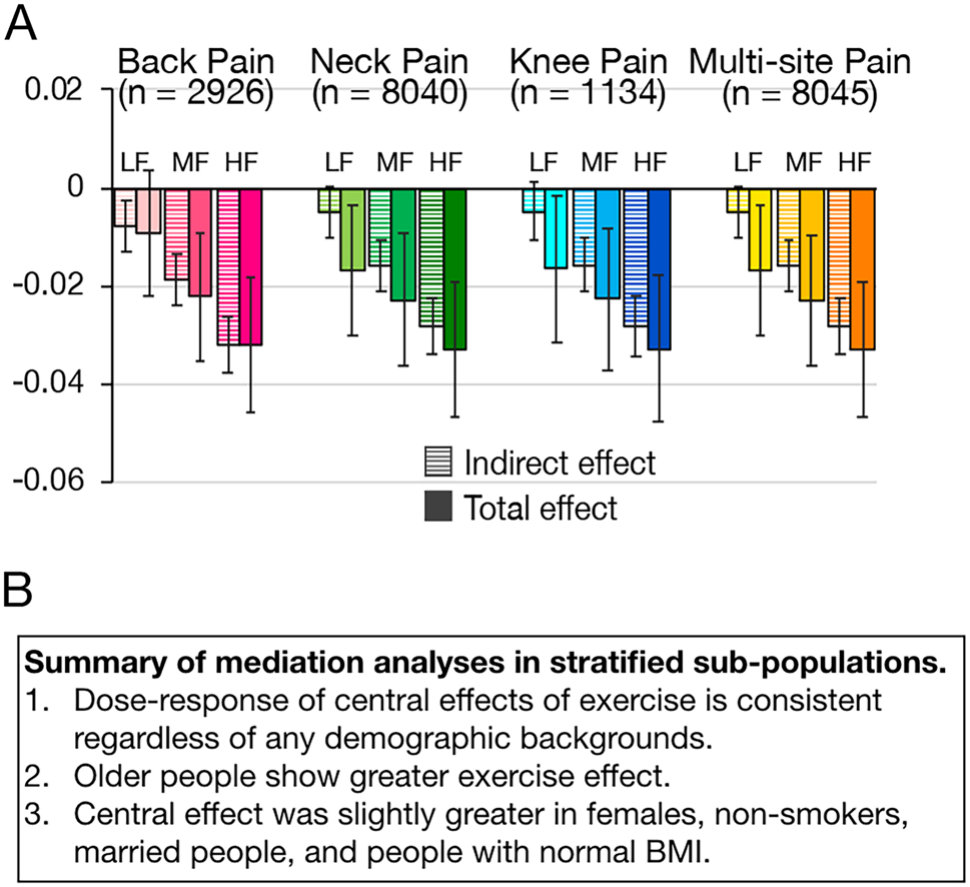
Stratified sub-populational analyses for the mediation model of central effects on exercise-related pain reduction.** (A)** Both indirect and total effects increased in parallel with the frequency of exercise in individuals with low back, neck, knee, and multi-site pain. The indirect effects showed at least 85% and 69% of the total effects of high- and moderate-frequency exercise respectively. **(B)** Summary table of the mediation analyses applied to the stratified subpopulations of demographic characteristics. Bootstrap mediation analyses were performed with 10,000 permutations, with adjustment for age, sex, BMI, low education, smoking status, marital status, living alone, living area, sleep duration, and pain duration. Error bars represent 95% confidence intervals. LF: low frequency, MF: moderate frequency, HF: high frequency.

Of 40,660 participants with pain, 2,926 (7.2%), 8,040 (19.8%), and 1,134 (2.9%) reported pain at only one of the three popular pain sites: low back, neck, and knees. 8,045 (19.8%) people reported pain at all three sites and were categorized as people with multi-site pain. The participants experiencing low back, neck, knee, as well as multi-site pain showed consistent dose-dependent responses (Fig. 3A and Supplementary Table 5). Full association was also demonstrated in all of them in terms of high- and moderate-frequency exercise.

Stratified mediation analyses identified an increasing tendency of the effect of exercise on pain reduction in parallel with the increasing age (Fig. 3B and Table 4). Especially, significant indirect as well as total effects of high- and moderate-frequency exercise were observed in participants aged > 40 years. Another important finding was that the significant indirect effects of high- and moderate-frequency exercise were consistent across all stratified populations. On the other hand, there were no significant total effects of exercise in younger people (aged < 40 years), current smokers, and people with chronic pain (pain duration ≥ 3 months).

**Table 4:** Sub-population analyses of the mediation model of the effect of exercise on pain intensity.

Subgroup (*n*)			RE (*n*)	Cumulative indirect effect					Total effect				
				Std-β	95% CI (LL, UL)	SD	*t*-value	*p*-value	Std-β	95% CI (LL, UL)	SD	*t*-value	*p*-value
Age (years)	20–29	(9763)	High (1619)	− 0.010	(− 0.019, − 0.001)	0.005	− 2.11	0.035	0.010	(− 0.015, 0.035)	0.013	0.79	0.428
			Mod (1076)	− 0.007	(− 0.015, 0.001)	0.004	− 1.64	0.100	0.012	(− 0.011, 0.036)	0.012	1.01	0.314
			Low (1033)	− 0.004	(− 0.013, 0.005)	0.004	− 0.92	0.357	0.000	(− 0.022, 0.023)	0.012	0.03	0.973
	30–39	(13,001)	High (2129)	− 0.023	(− 0.031, − 0.014)	0.004	− 5.25	< 0.001	0.006	(− 0.014, 0.026)	0.010	0.58	0.563
			Mod (1364)	− 0.011	(− 0.019, − 0.003)	0.004	− 2.80	0.005	0.004	(− 0.015, 0.023)	0.010	0.40	0.689
			Low (1145)	− 0.001	(− 0.008, 0.007)	0.004	− 0.14	0.889	0.001	(− 0.019, 0.020)	0.010	0.08	0.938
	40–49	(11,834)	High (1940)	− 0.033	(− 0.042, − 0.025)	0.004	− 7.82	< 0.001	− 0.047	(− 0.067, − 0.028)	0.010	− 4.82	< 0.001
			Mod (1131)	− 0.022	(− 0.030, − 0.014)	0.004	− 5.62	< 0.001	− 0.043	(− 0.062, − 0.024)	0.010	− 40.49	< 0.001
			Low (869)	− 0.001	(− 0.009, 0.007)	0.004	− 0.26	0.792	− 0.020	(− 0.039, − 0.001)	0.010	− 2.10	0.036
	50–59	(11,754)	High (2494)	− 0.029	(− 0.038, − 0.021)	0.004	− 7.07	< 0.001	− 0.048	(− 0.067, − 0.028)	0.010	− 4.85	< 0.001
			Mod (1167)	− 0.021	(− 0.028, − 0.015)	0.004	− 6.06	< 0.001	− 0.025	(− 0.044, − 0.006)	0.010	− 2.60	0.009
			Low (907)	− 0.009	(− 0.017, − 0.002)	0.004	− 2.37	0.018	− 0.008	(− 0.027, 0.011)	0.010	− 0.81	0.416
	60–64	(5349)	High (1627)	− 0.052	(− 0.064, − 0.040)	0.006	− 8.30	< 0.001	− 0.076	(− 0.107, − 0.046)	0.016	− 4.86	< 0.001
			Mod (635)	− 0.018	(− 0.029, − 0.007)	0.006	− 3.11	0.002	− 0.065	(− 0.094, − 0.036)	0.015	− 4.37	< 0.001
			Low (454)	− 0.017	(− 0.028, − 0.006)	0.006	− 2.94	0.003	− 0.038	(− 0.066, − 0.010)	0.014	− 2.64	0.008
Gender	Women	(25,779)	High (4217)	− 0.035	(− 0.041, − 0.029)	0.003	− 11.06	< 0.001	− 0.026	(− 0.040, − 0.012)	0.007	− 3.64	< 0.001
			Mod (2332)	− 0.018	(− 0.024, − 0.013)	0.003	− 6.57	< 0.001	− 0.006	(− 0.020, 0.007)	0.007	− 0.89	0.373
			Low (1944)	− 0.009	(− 0.015, − 0.004)	0.003	− 3.28	0.001	− 0.016	(− 0.029, − 0.003)	0.007	− 2.34	0.019
	Male	(25,922)	High (5592)	− 0.025	(− 0.031, − 0.020)	0.003	− 9.33	< 0.001	− 0.038	(− 0.051, − 0.025)	0.007	− 5.54	< 0.001
			Mod (3041)	− 0.016	(− 0.021, − 0.011)	0.002	− 6.41	< 0.001	− 0.039	(− 0.052, − 0.027)	0.006	− 6.08	< 0.001
			Low (2464)	− 0.003	(− 0.008, 0.002)	0.003	− 1.29	0.196	− 0.008	(− 0.021, 0.004)	0.006	− 1.31	0.192
BMI (kg/m^2^)	< 20	(12,964)	High (2260)	− 0.029	(− 0.036, − 0.021)	0.004	− 7.08	< 0.001	− 0.038	(− 0.056, − 0.019)	0.009	− 4.01	< 0.001
			Mod (1182)	− 0.014	(− 0.020, − 0.007)	0.003	− 3.89	< 0.001	− 0.016	(− 0.034, 0.001)	0.009	− 1.81	0.070
			Low (963)	− 0.006	(− 0.012, 0.001)	0.003	− 1.61	0.108	− 0.015	(− 0.032, 0.002)	0.009	− 1.74	0.082
	≥ 20, < 25	(25,730)	High (5284)	− 0.034	(− 0.039, − 0.028)	0.003	− 11.64	< 0.001	− 0.033	(− 0.047, − 0.020)	0.007	− 4.86	< 0.001
			Mod (2842)	− 0.020	(− 0.025, − 0.015)	0.003	− 7.50	< 0.001	− 0.025	(− 0.038, − 0.012)	0.007	− 3.83	< 0.001
			Low (2283)	− 0.007	(− 0.012, − 0.001)	0.003	− 2.44	0.015	− 0.014	(− 0.028, − 0.001)	0.007	− 2.17	0.030
	≥ 25	(9607)	High (1607)	− 0.024	(− 0.033, − 0.015)	0.004	− 5.36	< 0.001	− 0.023	(− 0.044, − 0.001)	0.011	− 2.07	0.038
			Mod (969)	− 0.015	(− 0.023, − 0.007)	0.004	− 3.67	< 0.001	− 0.024	(− 0.044, − 0.004)	0.010	− 2.35	0.019
			Low (873)	− 0.006	(− 0.014, 0.002)	0.004	− 1.47	0.142	− 0.003	(− 0.024, 0.018)	0.011	− 0.26	0.795
Education	Low	(30,225)	High (5256)	− 0.029	(− 0.034, − 0.024)	0.003	− 10.79	< 0.001	− 0.035	(− 0.048, − 0.023)	0.006	− 5.48	< 0.001
			Mod (2679)	− 0.018	(− 0.023, − 0.014)	0.002	− 7.65	< 0.001	− 0.034	(− 0.046, − 0.022)	0.006	− 5.63	< 0.001
			Low (2318)	− 0.006	(− 0.011, − 0.002)	0.003	− 2.59	0.010	− 0.014	(− 0.026, − 0.002)	0.006	− 2.27	0.023
	High	(21,476)	High (4553)	− 0.032	(− 0.038, − 0.025)	0.003	− 9.43	< 0.001	− 0.027	(− 0.043, − 0.012)	0.008	− 3.48	< 0.001
			Mod (2694)	− 0.016	(− 0.022, − 0.010)	0.003	− 5.29	< 0.001	− 0.007	(− 0.022, 0.008)	0.008	− 0.92	0.356
			Low (2090)	− 0.006	(− 0.012, 0.000)	0.003	− 2.11	0.035	− 0.011	(− 0.026, 0.004)	0.008	− 1.45	0.148
Smoking	Current	(12,731)	High (2321)	− 0.024	(− 0.032, − 0.016)	0.004	− 5.94	< 0.001	− 0.019	(− 0.038, 0.000)	0.010	− 1.92	0.055
			Mod (1277)	− 0.017	(− 0.024, − 0.009)	0.004	− 4.56	< 0.001	− 0.002	(− 0.021, 0.017)	0.010	− 0.25	0.805
			Low (1011)	− 0.016	(− 0.023, − 0.009)	0.004	− 4.31	< 0.001	− 0.019	(− 0.037, − 0.001)	0.009	− 2.07	0.039
	Non	(38,970)	High (7488)	− 0.033	(− 0.038, − 0.028)	0.002	− 13.44	< 0.001	− 0.039	(− 0.050, − 0.027)	0.006	− 6.71	< 0.001
			Mod (4096)	− 0.018	(− 0.022, − 0.013)	0.002	− 8.08	< 0.001	− 0.030	(− 0.041, − 0.019)	0.006	− 5.47	< 0.001
			Low (3397)	− 0.003	(− 0.008, 0.001)	0.002	− 1.49	0.135	− 0.011	(− 0.022, − 0.001)	0.005	− 2.06	0.040
Living	Alone	(9496)	High (1986)	− 0.032	(− 0.042, − 0.022)	0.005	− 6.31	< 0.001	− 0.035	(− 0.058, − 0.012)	0.012	− 2.97	0.003
			Mod (1079)	− 0.018	(− 0.027, − 0.010)	0.004	− 4.25	< 0.001	− 0.023	(− 0.045, − 0.001)	0.011	− 2.05	0.041
			Low (869)	− 0.004	(− 0.013, 0.006)	0.005	− 0.74	0.457	− 0.016	(− 0.036, 0.004)	0.010	− 1.55	0.122
	Family	(42,205)	High (7823)	− 0.030	(− 0.034, –0.025)	0.002	–12.54	< 0.001	–0.032	(–0.043, –0.021)	0.006	–5.72	< 0.001
			Mod (4294)	–0.017	(− 0.021, − 0.013)	0.002	− 8.46	< 0.001	− 0.023	(− 0.033, − 0.013)	0.005	− 4.53	< 0.001
			Low (3539)	− 0.007	(− 0.011, − 0.003)	0.002	− 3.37	< 0.001	− 0.012	(− 0.022, − 0.002)	0.005	− 2.29	0.022
Marital	Married	(28,355)	High (5426)	− 0.034	(− 0.039, − 0.028)	0.003	− 11.05	< 0.001	− 0.030	(− 0.044, − 0.017)	0.007	− 4.41	< 0.001
			Mod (2982)	− 0.018	(− 0.023, − 0.013)	0.003	− 7.30	< 0.001	− 0.023	(− 0.036, − 0.010)	0.007	− 3.49	< 0.001
			Low (2343)	− 0.007	(− 0.012, − 0.002)	0.003	− 2.69	0.007	− 0.009	(− 0.021, 0.004)	0.006	− 1.32	0.186
	Single	(23,346)	High (4383)	− 0.025	(− 0.032, − 0.019)	0.003	− 8.25	< 0.001	− 0.033	(− 0.048, − 0.019)	0.007	− 4.57	< 0.001
			Mod (2391)	− 0.015	(− 0.021, − 0.010)	0.003	− 5.47	< 0.001	− 0.021	(− 0.035, − 0.006)	0.007	− 2.81	0.005
			Low (2065)	− 0.005	(− 0.011, 0.000)	0.003	− 1.85	0.065	− 0.018	(− 0.031, − 0.004)	0.007	− 2.59	0.010
Pain duration	< 3 M	(22,059)	High (4228)	− 0.035	(− 0.040, − 0.030)	0.003	− 13.48	< 0.001	− 0.040	(− 0.052, − 0.029)	0.006	− 6.83	< 0.001
			Mod (2328)	− 0.018	(− 0.023, − 0.014)	0.002	− 7.83	< 0.001	− 0.030	(− 0.041, − 0.019)	0.006	− 5.44	< 0.001
			Low (1860)	− 0.004	(− 0.009, 0.000)	0.002	− 1.77	0.077	− 0.014	(− 0.025, − 0.003)	0.006	− 2.46	0.014
	≥ 3 M	(18,601)	High (3375)	− 0.025	(− 0.033, − 0.016)	0.004	− 5.74	< 0.001	− 0.017	(− 0.037, 0.003)	0.010	− 1.69	0.091
			Mod (1975)	− 0.018	(− 0.025, − 0.010)	0.004	− 4.50	< 0.001	− 0.003	(− 0.022, 0.016)	0.010	− 0.33	0.742
			Low (1741)	− 0.017	(− 0.024, − 0.009)	0.004	− 4.25	< 0.001	− 0.019	(− 0.037, 0.000)	0.009	− 1.99	0.047

Dose-dependent significant exercise effects on pain through psychological components were consistent in individuals aged > 40 years, both males and females, of any weight categories: underweight (BMI < 20 kg/m^2^), normal weight (BMI ≥ 20, < 25 kg/m^2^), and overweight (BMI ≥ 25 kg/m^2^), individuals with both low and high education, non-smokers, individuals living alone and with family, both married and single individuals, and those with less than 3 months (3 M) duration of pain. The effect of exercise on pain was not identified in individuals aged < 40 years, current smokers, or individuals with persistent pain (pain duration ≥ 3 months). The effect of exercise increased with age. Bootstrap analysis was performed with 10,000 permutations, with adjustment for age, sex, BMI, low education, marital status, living alone, living area, smoking status, sleep duration, and pain duration, except for the variable of each sub-population. The cumulative indirect effect was a combination of the path effects of Negative Affect (*a*1 × *b*1) and Vigor (*a*2 × *b*2). RE: regular exercise, Std-β: standardized regression coefficient, CI: confidence interval, LL: lower limit, UL: upper limit, SD: standard deviation.

## Discussion

Several participants who mentioned having moderate-to-severe pain, had chronic pain (≥ 3 months) and multi-site pain indicating that these participants were more likely to have severe pain and a lower status of the overall subjective health. 16.9% of the participants with moderate-to-severe pain reported exercising at least twice a week, although this percentage was lower compared to participants without pain or those with mild pain, which is suggestive of the fact that people with more severe pain might have difficulty in establishing an exercise routine. However, the proportion of participants who exercised less than twice a week was not as low as those without pain, suggesting that these participants were more motivated to maintain an exercising habit. Additionally, the significant association of severe pain with risk factors such as women, high BMI, low education, smoking habits, stress and negative affect and short sleep was consistent with previous studies^16,17^.

The rate of high-frequency exercise was similar in participants with mild pain and those without pain, however, the rate of moderate-frequency and low-frequency exercise was higher in participants without pain, potentially resulting in higher scores on the vigor scale compared to those without pain. This suggests that the presence or absence of exercise habits in people with pain is associated with both pain intensity and emotion, and lack of exercise is not decided only by presence of pain.

The fact that two components, Negative affect (PC1) and Vigor (PC2), were extracted by PCA suggests that positive and negative emotions are not simply two sides of the same coin, but rather should be evaluated separately. This finding reinforces the importance of evaluating pain-related fear as well as functional self-efficacy, when implementing treatments for chronic low back pain patients^18^.

While pain is one aspect of subjective health, the fact that the mediation model in this study showed partial association with regards to subjective health (Supplementary Fig. 1), but full association with regards to pain intensity (Fig. 2A), indicates that the emotional effects of exercise on pain intensity are more prominent than those on subjective health. In other words, the association effects of exercise on subjective health may be controlled by other aspects such as physical improvement as well and not only the emotional aspect.

Although the effects of EIH depend on the type, amount, intensity of exercise, and the presence of pain during exercise, this study did not conduct the investigation of the detailed types of exercise and performed the analyses with heterogeneity in background. Unsupervised or voluntary exercise, whose proportion might be majority in this study, show small effect as a treatment for pain compared to supervised exercise therapy^19^. Therefore, we considered that our findings showed small amount of absolute standardized coefficients of exercise habit to pain intensity and small adjusted coefficient of determination in the model 1, Table 2. On the other hand, the fact that the significant associations of exercise habit disappeared in the model 2 made us come up with the mediation model of Negative affect (PC1) and Vigor (PC2). As a result, the full association model of Negative affect (PC1) and Vigor (PC2) was established and successfully validated with a dose-dependent response even for participants with pain in the low back, neck, knee, and pain in all three locations, indicating that the impact of exercise habits on emotional aspects may be an important universal point in the effect of exercise habits on pain regardless of the pain site. Nobel point of this study was the development of the full association model of exercise effect on pain, even though absolute values of coefficients were small. Our findings interpret that improvements of negative affect and positive one should be paid attention to in an exercise habit for people with pain.

According to our recently reported study of brain functional connectivity associated with exercise effect on pain^20^, exercise habit is associated with decreased functional connections in the left thalamus and right amygdala, and increased ones in the medial prefrontal cortex (MPFC). Thalamus plays the role of a central nucleus on the sensory pathway, and the amygdala and MPFC are involved in recognition of negative emotion and/or unpleasantness. Our findings in the mediation analyses might clinically correspond to these neurological modifications induced by exercise habit.

EIH, a consistent phenomenon of pain attenuation following exercise, is possibly an important factor of exercise-related pain reduction. Although the mechanisms responsible for EIH are not entirely understood^21^, central modifications, (e.g., serotonergic^22,23^, dopaminergic^24^, endocannabinoid^25,26^, and opioid systems^27^), and involvement of conditioned pain modulation through the descending pathways are thought to be the responsible factors^28^. The improvements in Negative affect (PC1) and Vigor (PC2) after exercise might be a result of these central mechanisms. On the other hand, people with chronic pain are generally associated with impairments of these systems^29^. Complex pathophysiology involving psychological factors and alterations in the central nervous system are the characteristics of chronic pain^30^. Therefore, although exercise therapy is an appropriate treatment for chronic pain, the effective extent of pain improvement is limited^31^. Similarly, in this study, the group with chronic pain tended to have a limited improvement in pain, leading to the belief that the impact of exercise on pain intensity was minimal.

This study indicated an increasing effect of exercise on pain relief with increasing age, suggesting the involvement of the endogenous pain inhibition mechanisms, that decrease in function with age^32^. However, this function is reversible. A previous study investigating central sensitization and the descending pain inhibitory system using quantitative sensory testing in older adults has demonstrated that those with higher physical activity levels have better functioning pain inhibition mechanisms^33^. Such biological mechanisms may lead to differences by age group in pain relief responsiveness.

Women are generally associated with increased pain sensitivity, lower pain threshold, and increased risk of developing clinical pain, as compared to men^34,35^. On the other hand, although gender differences with respect to response to pain treatment have not been clearly understood, few reports suggest that women respond better to interdisciplinary treatment compared to men, and that gender is a factor that is related to responsiveness to pain treatment^36^. The results of this study also suggest that women may have a higher tendency for the psychological factors of exercise to influence pain intensity compared to men.

This study has also indicated that married people tended to have higher indirect effects of Negative affect (PC1) and Vigor (PC2) on pain intensity in relation to exercise, compared to unmarried people. However, according to previous studies, the presence or absence of a spouse does not affect unpleasantness or suffering related to pain^37^, and is not a determining or predictive factor for quality of life, and therefore, need not be considered during rehabilitation^38^. Therefore, the effects of marital status on exercise and pain needs to be further investigated.

Severity of chronic pain is affected by lifestyle factors such as smoking and high body weight^39^. Studies investigating patients with lumbar disc herniation have identified that smoking and high body weight are risk factors for motor deficits and delayed pain improvement^40^. In fact, smoking and high body weight have been shown to adversely affect responsiveness to exercise therapy on treatment^41,42^, which is consistent with our findings that the total effect of exercise habits on pain was lesser in people with BMI ≥ 25 kg/m^2^ or in people who practiced smoking.

Some limitations of the study need to be addressed. Firstly, the nature of the web-based survey may reduce the external validity of the results because access to the internet is necessary for the online recruitment. However, the study was conducted with a large sample size, corresponding to the general population in Japan, in terms of age and sex composition ratio. Therefore, the selection bias may not be a critical problem in this study. Secondly, the influence of pain intensity on exercise frequency was not assessed, although a bidirectional causal relationship may be present between these two factors. In this study, participants reported exercise frequency over the past year, and the pain intensity reported was over the past four weeks. Therefore, the directionality from pain intensity to exercise habit could not be considered. Thirdly, the detailed properties of exercise were not assessed in the study. Data for the duration, intensity, and type of exercise was not collected, since we assumed that these parameters were optimized by people who exercise. From the viewpoint of exercise optimization, exercise therapy supervised by a professional therapist is beneficial^19^. Fourthly, subjective stress and health were assessed by an original measurement without scientific validation. However, the 11-point numerical rating scale that we used is a measurement widely used for assessing a single item and convenient to assess it for many people in a limited time. Although further confirmations may be required for our findings, this data collection way for subjective stress and health is considered to be acceptable for scientific researches.

Fifthly, a recall bias can potentially affect the retrospective questions. Finally, only adults under 65 years old were included in this study and the effects in the elderlies are still unclear. Although greater effects according to aging are expected from the findings in the subpopulation analyses of the age category, further study is required to identify them in elderlies. Thus, the responses should be interpreted with caution.

In conclusion, this study has demonstrated that the effect of exercise on pain reduction is associated with psychological components, namely Negative Affect and Vigor. These association effects increased in parallel with the frequency of exercise habit. Furthermore, the full mediation model with a dose-dependent response was successfully validated regardless of the pain site, suggesting improvement of the negative and positive emotion is comprehensive factor of the exercise effect on pain.

### Supplementary Information

Supplementary Information.

## Data Availability

Data are available upon reasonable request. Analyzed data in this study are considered to be available under the permission of the corresponding author and data manager.
