# Supplementary material for: Exercise effect on pain is associated with negative and positive affective components: A large-scale internet-based cross-sectional study in Japan
Source: Sci Rep. 2024 Apr 1;14:7649. doi: 10.1038/s41598-024-58340-z (PMC10985089; doi:10.1038/s41598-024-58340-z)
Supplement: Supplementary file 1 — Supplementary Information. [file 41598_2024_58340_MOESM1_ESM.docx]

**Supplementary Materials**


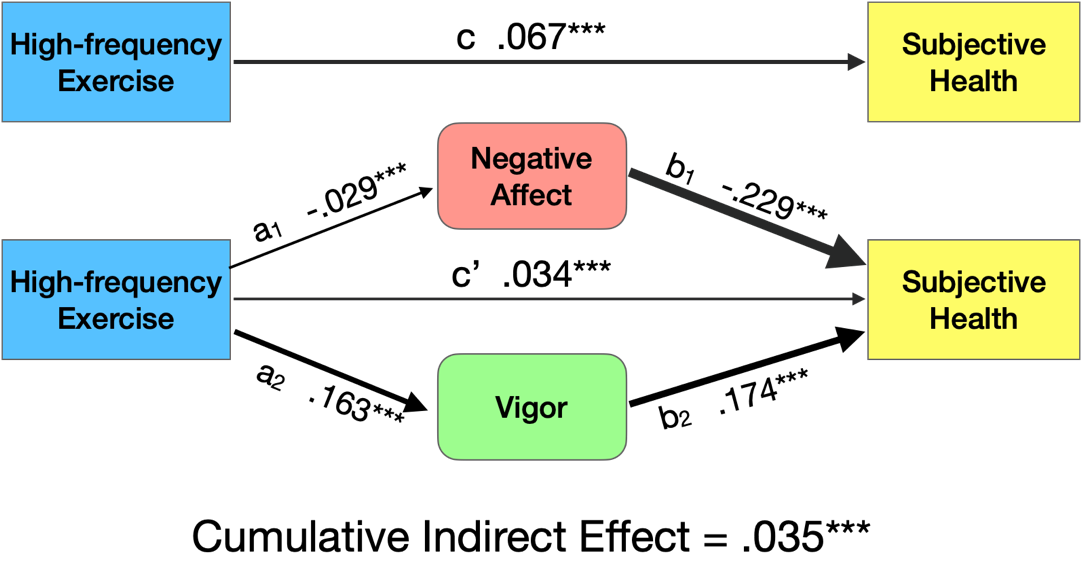


**Supplementary Figure 1. Partial mediation model of the effect of high-frequency exercise on subjective health through psychological components in the Discovery group (n = 20,330).** The mediation analysis for subjective health identified significant cumulative indirect (0.035, 95% confidence interval [CI]; 0.030 to 0.040) and direct effects (0.34, 95% CI; 0.021 to 0.048), suggesting a partial association (association proportion = 0.52). The thickness of the path represents the absolute value of the effect; ***p < 0.001

**Supplementary Table 1. Bootstrap multivariable mediation analysis for the effect of exercise habits on pain intensity through psychological components in the Discovery group (n = 20,330).** High-, moderate-, and low-frequency exercise showed significant indirect effects. The direct effect was nearly zero, and the cumulative indirect effect was nearly equal to the total effect observed in high-frequency exercise (association proportion = 0.99), suggesting a full association model. Furthermore, dose-dependent responses were identified in the total and indirect effects, as well as exercise effects on each psychological component, corresponding to the frequency of exercise habit. Bootstrap analysis was performed with 10,000 permutations, under control for age, sex, BMI, low education, marital status, living alone, living area, smoking status, sleep duration, and pain duration. The indirect effect was a combination of the path effects of Negative Affect (a_1_ × b_1_) and Vigor (a_2_ × b_2_). Std-β: standardized regression coefficient, CI: confidence interval, LL: lower limit, UL: upper limit, SD: standard deviation.

**Supplementary table 2. Bootstrap multiple mediation analysis for effect of exercise habits on pain intensity through psychological components in Validation (n = 20,330).** High- and moderate-frequency exercise showed significant indirect effects and insignificant direct effects, indicating replicated full association. Association proportion of the high-frequency exercise was 0.85. Dose-dependent responses were also consistent in the total and indirect effects as well as exercise effects on each psychological component corresponding to the frequency of exercise habit. Bootstrap analysis was performed with 10,000 permutations, with adjustments of age, sex, BMI, low education, marital status, living alone, living area, smoking, sleep duration, and pain duration. Indirect effect was combined of the path effects of Negative Affect (a_1_ × b_1_) and Vigor (a_2_ × b_2_). Std-β: standardized regression coefficient, CI: confidence interval, LL: lower limit, UL: upper limit, SD: standard deviation.

**Supplementary table 3. Bootstrap multivariable mediation analysis for effect of exercise habits on subjective health through psychological components in Discovery (n = 20,330).** The mediation analysis of high-frequency exercise identified significant cumulative indirect and direct effects, suggesting a partial association (association proportion = 0.52). Although dose-dependent responses were consistent to the association model for pain intensity, full association was not identified. Bootstrap analysis was performed with 10,000 permutations, with adjustments of age, sex, BMI, low education, marital status, living alone, living area, smoking, sleep duration, pain duration, and pain intensity. Indirect effect was combined of the path effects of Negative Affect (a_1_ × b_1_) and Vigor (a_2_ × b_2_). Std-β: standardized regression coefficient, CI: confidence interval, LL: lower limit, UL: upper limit, SD: standard deviation.

**Supplementary Table 4. Difference of path effects of exercise habits on pain intensity compared with subjective health in the Discovery group (n = 20,330).** The absolute values of path effects were compared between the association model for pain intensity and that for subjective health. In terms of high- and moderate-frequency exercise, significant differences were identified in the indirect path effects of Negative affect and Vigor, whereas the cumulative indirect effects were not statistically different. In contrast, the direct and total effects of high-frequency exercise showed a significant difference. Bootstrap analysis was performed with 10,000 permutations. The indirect effect was a combination of the path effects of Negative Affect (a_1_ × b_1_) and Vigor (a_2_ × b_2_). Δstd-β: difference of absolute standardized regression coefficient (pain intensity > subjective health), CI: confidence interval, LL: lower limit, UL: upper limit, SD: standard deviation.

**Supplementary Table 5. Bootstrap multivariable mediation analyses for the effect of exercise habits on pain intensity through the psychological components in four sub-populations of individuals with back, neck, knee, and multi-site pain.** In terms of high- and moderate-frequency exercise, dose-dependent responses of the exercise effects and full association effects of the psychological components were consistent across all sub-populations, as indicated by significant indirect effects and insignificant direct effects. Bootstrap analysis was performed with 10,000 permutations, with adjustments for age, sex, BMI, low education, marital status, living alone, living area, smoking status, sleep duration, and pain duration. The indirect effect was a combination of the path effects of Negative Affect (a_1_ × b_1_) and Vigor (a_2_ × b_2_). Std-β: standardized regression coefficient, CI: confidence interval, LL: lower limit, UL: upper limit, SD: standard deviation.
